# Damage Identification in Plate Structures Using Sparse Regularization Based Electromechanical Impedance Technique

**DOI:** 10.3390/s20247069

**Published:** 2020-12-10

**Authors:** Xingyu Fan, Jun Li

**Affiliations:** 1 Guangzhou University-Curtin University Joint Research Centre for Structural Monitoring and Protection against Multi-Dynamic Hazards, School of Civil Engineering, Guangzhou University, Guangzhou 510006, China; xingyu.fan@graduate.curtin.edu.au; 2 Centre for Infrastructural Monitoring and Protection, School of Civil and Mechanical Engineering, Curtin University, Perth 6102, WA, Australia

**Keywords:** damage quantification, plate structures, impedance, sparse regularization, resonance frequency shifts, undetermined inverse problem

## Abstract

This paper proposes a novel structural damage quantification approach using a sparse regularization based electromechanical impedance (EMI) technique. Minor structural damage in plate structures by using the measurement of only a single surface bonded lead zirconate titanate piezoelectric (PZT) transducer was quantified. To overcome the limitations of using model-based EMI based methods in damage detection of complex or relatively large-scale structures, a three-dimensional finite element model for simulating the PZT–structure interaction is developed and calibrated with experimental results. Based on the sensitivities of the resonance frequency shifts of the impedance responses with respect to the physical parameters of plate structures, sparse regularization was applied to conduct the undetermined inverse identification of structural damage. The difference between the measured and analytically obtained impedance responses was calculated and used for identification. In this study, only a limited number of the resonance frequency shifts were obtained from the selected frequency range for damage identification of plate structures with numerous elements. The results demonstrate a better performance than those from the conventional Tikhonov regularization based methods in conducting inverse identification for damage quantification. Experimental studies on an aluminum plate were conducted to investigate the effectiveness and accuracy of the proposed approach. To test the robustness of the proposed approach, the identification results of a plate structure under varying temperature conditions are also presented.

## 1. Introduction

Owing to the needs for damage detection of structures, numerous non-destructive evaluation (NDE) based structural health monitoring (SHM) methods have been developed and successfully applied in recent years [1,2,3,4,5,6]. NDE methods, such as radar [7], ultrasonic [8] and impact-echo [9] based non-destructive testing (NDT), have become available and have been widely applied for condition assessment and evaluation of civil engineering structures. Even though such techniques show promising performance in practical applications, the high cost of NDT equipment, the requirement for specific experience and knowledge of the operators, and the limited region that these techniques can examine may narrow their applications and increase the labor cost for structural inspection. Meanwhile, piezoelectric (PZT) material has been widely used in local SHM methods, due to its lightweight nature, robustness to the environment and cost-effectiveness [10]. With these advantages, PZT transducers can be used to interact with structures and generate electromechanical signatures when the host structure is subjected to high frequency excitations. These electromechanical signatures can be used to monitor structural conditions. This method is usually named as the electromechanical impedance (EMI) based method. Compared with the traditional global SHM methods based on the modal information, such as nature frequencies and mode shapes, the EMI method is much more sensitive to structural damage at a smaller scale because of its high working frequency [10].

EMI methods have been developed to detect the existence of structural damage based on the change in the measured EMI behaviour with PZT transducers. Based on defined damage indicators, EMI based damage detection methods can be generally divided into two different categories: non-model based EMI methods and model based EMI methods.

For non-model based EMI methods, the occurrence of structural damage is detected by simply evaluating the difference between measured EMI signatures from the pristine and damaged states of the monitored structure. To quantitatively estimate this difference between the impedance signatures, several statistical damage indicators have been developed. Sun, et al. [11] presented a frequency domain impedance signature based damage detection method using root mean square deviation (RMSD) to describe such difference. Later, the correlation coefficient (CC) between the impedance signatures was calculated to reflect the damage severity by Raju [12]. Tseng and Naidu [13] compared the performance of using non-parametric damage indicators, namely, RMSD, CC, mean absolute percentage deviation (MAPD) and covariance (Cov), for EMI based monitoring with experimental investigations on aluminum specimens. It has been found that RMSD shows a higher sensitivity to the new damage occurrence than the other three metrics. Besides these frequency domain based analysis methods, other more complex non-parametric metrics have been investigated based on the input and output time-series analysis measured by PZT transducers [14,15,16]. Nevertheless, to improve the performance of using EMI methods with non-parametric metrics in damage detection, machine learning algorithms based EMI damage identification methods have also been developed for different damage scenarios [17,18,19,20]. Although such frequency domain impedance signature based damage metrics have been successfully developed for monitoring the conditions of practical structures [21,22,23,24], the non-parametric metrics based methods have been developed to evaluate the impedance signatures through the phenomenological characteristics. The accurate localization and quantification of structural damage remains a significant challenge. For damages of the same severity, the value of the statistical indicator only depends on the distance between the damage and the PZT transducer. To distinguish the damages at different locations that have the same distance from the measurement location, artificial intelligence methods based on EMI techniques have been developed. The impedance based damage detection methods have been incorporated with neural network methods [17,18]. However, the main drawback of the aforementioned applications is that the impedance responses of multiple PZT transducers at various locations are necessary. Moreover, training of the neural networks is still based on the evaluation of phenomenological characteristics of the impedance signatures, which is not explicitly related to the physical properties of the host structure. Therefore, the selected frequency range and neural networks model is not be suitable for different structures or different damages.

To demonstrate the relationship between the electromechanical impedance response and structural mechanical prosperities, such as stiffness, mass and damping, accurate simulation for the interaction between a PZT transducer and the host structure is necessary. Analytical modelling methods for PZT–structure interactions have been developed [25,26,27,28]. Even though these models can provide the analytical expression of the relationship between the EMI and mechanical impedance of a structure, it is still difficult to predict the accurate impedance behaviours of structures using the analytical methods, especially for relatively large scale structures, e.g., plates. To overcome this drawback, numerical models for EMI based SHM techniques have been developed to simulate the EMI behavior of the surface bonded PZT actuator attached on structures [29,30,31,32].

Taking advantage of the accurate finite element modelling, sensitivity analyses have been employed in model based EMI damage identification methods for inverse problems. Rayleigh–Ritz and spectral element modelling methods have been used in the model based EMI methods owning to their high computational efficiency. Instead of evaluating the impedance signatures by non-parametric metrics for damage detection, alternatively, model based EMI methods can be developed to identify the location and severity of structural damages by updating the physical parameters of the numerical model of the host structure with surface bonded PZT transducers based on measured impedance responses. Xu [33] investigated the general impedance model and developed a damage detection method using evolutionary programming. However, there was a non-negligible deviation between the measured impedance signature and the simulation result of the generic two-dimensional (2D) model. Investigations were carried out to enhance the prediction accuracy and computational efficiency of the optimization method by using pre-screening and statistical calibration techniques to reduce the search space and obtain more accurate impedance prediction with finite element analysis of PZT–structure interaction systems [34,35]. By taking advantage of computational inverse techniques, EMI based damage identification methods using sensitivity based model updating have been developed. Due to the lower computational cost compared to the conventional finite element model, the spectral element model has been employed for EMI based damage identification methods [36,37]. Wang and Tang [38] developed a spectral element model to identify both the location and severity of structural damage. The effectiveness of the proposed method was verified by experimental studies. Nevertheless, this 2D numerical modelling method with a high computational accuracy and efficiency is only applicable to simple structures, i.e., beams, based on the nature of spectral element methods. The aforementioned experimental study with spectral element modelling was conducted on a narrow beam which has a similar width to the bonded PZT transducer. Later on, a three-dimensional spectral finite element method was developed by Sepehry, et al. [39] based on the discrete singular convolution method. Numerical results showed a good agreement with the experimental results for a narrow beam. A significant difference was observed between the simulation and experimental results for a plate structure.

On the other hand, using the conventional Tikhonov regularization method to solve the ill-conditioned inverse problem for damage localization and identification is limited by the major drawback that the solution tends to be overly smooth [40]. In terms of structural damage identification, this means that the predicted damages tend to distribute in most of the structural segments around the true damage location. This trend contradicts with the real structural damage which usually occurs at a few locations. Recently, sparse regularization (or the *l*_1_ norm regularization technique) based damage identification method has been developed to overcome the abovementioned limitation [41,42]. Cao, et al. [43] developed the EMI based structural damage detection method using model updating incorporated with the *l*_0_ norm regularization technique. The multi-objective Dividing RECTangles (DIRECT) algorithm was implemented to solve the multi-objective optimization problem. The admittance (inverse of impedance) of 21 frequency points around two impedance resonant peaks were used to update the finite element model and identify the potential structural damage in 25 segments of an aluminum beam. Fan, et al. [44] developed a novel method for structural damage identification based on impedance response sensitivity and *l_1_* norm regularization technique. Experimental studies on beam structures were conducted to validate the accuracy of using the developed method to identify the location and severity of structural damage, with only a limited number of resonant frequencies in impedance responses (seven resonance frequency shift measurements for 30 potential fault candidates). Conducting the modeling to simulate the PZT–structure interaction in large scale structures is challenging, e.g., for plate structures, since a very fine mesh is required for structural modeling to obtain accurate impedance responses in a very high frequency range. Therefore, a very large number of elements will be observed for a large size structural model. This brings significant difficulty for model based structural damage identification, since the number of unknown parameters will become much larger than that of the available measurements. However, the available and effective features from impedance response data are limited. To the best of authors’ knowledge, the sparse regularization based EMI technique has not been extended to structural damage quantification in plate like structures.

For current non-model based EMI methods, the main limitation is the difficulty to quantify and locate structure damage. Even though model-based EMI techniques have been carried out to overcome this drawback, most of the previous experimental studies of such method were carried out on the simple beam structures.

In this paper, a new EMI based structural damage quantification approach, using model updating with impedance sensitivity and the sparse regularization technique, is developed. Based on the modelling method shown in the previous work, the proposed sparse regularization based damage identification method is extended to the plate structure, which has been rarely studied with the EMI technique. The sensitivity of resonance frequency shifts in impedance behaviors with respect to structural stiffness parameters are used to update the finite element model of the host structure and identify the stiffness reductions in the host structure that are related with structural damage. Experimental studies on an aluminum plate with a surface bonded PZT transducer are conducted to validate the accuracy and performance of the proposed approach for structural damage quantification in relatively large structures, e.g., plates. The sparse regularization technique is applied to achieve accurate identification results for the host structure with numerous segments, but using a limited amount of measured impedance resonance frequency shifts. Compared with the study on a beam structure [44], the number of unknown structure parameters is significantly increases which also greatly increased the difficulty in solving the inverse damage identification problem.

## 2. Finite Element Modelling for EMI Based SHM Technique

### 2.1. Background

In this study, the finite element model of simulating PZT-structure interaction is developed by using COMSOL Multiphysics 5.2. Piezoelectric analysis is considered as the multi-physics field analysis, which describes the coupling effect between the mechanical and electric properties of piezo-material. Based on the combination of the linear constitution relationship of electromechanical coupling effect by Yang, et al. [29], the coupled interaction equation can be expressed as

(1) $$\left[\begin{array}{cc} \left[M\right] & 0\\ 0 & 0 \end{array}\right]\left\{\begin{array}{c} \left\{\ddot{u}\right\}\\ \left\{\ddot{V}\right\} \end{array}\right\}+\left[\begin{array}{cc} \left[C\right] & 0\\ 0 & 0 \end{array}\right]\left\{\begin{array}{c} \left\{\dot{u}\right\}\\ \left\{\dot{V}\right\} \end{array}\right\}+\left[\begin{array}{cc} \left[K\right] & \left[K^{Z}\right]\\ {\left[K^{Z}\right]}^{T} & \left[K^{d}\right] \end{array}\right]\left\{\begin{array}{c} \left\{u\right\}\\ \left\{V\right\} \end{array}\right\}=\left\{\begin{array}{c} \left\{F\right\}\\ \left\{L\right\} \end{array}\right\}$$

where [M], [C] and [K] are the mass, damping and stiffness matrices of structural properties, respectively; [u] denotes the nodal displacement vector; {V} is the electric potential vector; {F} and {L} are the vectors of force and electrical charge, respectively; $\left[K^{Z}\right]$ is the piezoelectric coupling matrix and $\left[K^{d}\right]$ is the dielectric conductivity. In COMSOL Multiphysics, the electrodes current $\bar{I}$ is calculated by a surface integral of the inward current, and the voltage $\bar{V}$ at the same electrode is defined by boundary conditions. Then the complex impedance signature can be expressed as

(2) $$\bar{Y}=\frac{\bar{V}}{\bar{I}}$$

### 2.2. Damping Model

One of the most commonly used damping models in previous studies is the Rayleigh damping model, which can be expressed as

(3) C=αM+βK

where α and β are the damping model coefficients for mass and stiffness matrices. These multipliers can be obtained by the following equation

(4) $$\zeta_{r}=\frac{\alpha }{2\omega_{r}}+\frac{\beta \omega_{r}}{2}$$

By using the first two orders of structural modes, the exact value of the damping coefficients can be calculated. In Equation (4), $\omega_{r}$ and $\zeta_{r}$ denote the $r^{th}$ order of the natural frequency and damping ratio, respectively. In the existing studies, the damping coefficient α which is related to the mass matrix, is assumed as zero [29], and the damping model will be only decided by the second term of Equation (3). Then Equation (4) can be rewritten as

(5) $$\beta_{r}=\frac{2\zeta_{r}}{\omega_{r}}$$

Owing to a wide frequency range of the excitation used in the EMI based SHM techniques, a finite element model using a weaker frequency dependent damping model, namely, the hysteretic damping model, has been developed by Lim and Soh [45].

The conventional Rayleigh damping model shows a non-negligible limitation in simulating impedance responses for the large and high frequency range. Since $\beta_{r}$ is a constant value, the use of Rayleigh damping means that the modes with a resonance frequency larger than $\frac{2}{\beta_{r}}$ will be overdamped. Therefore, the hysteretic damping model, which is frequency independent and weakly related with frequency, can be more accurate for the EMI prediction. For hysteretic damping, a variable stiffness multiplier is used as follows

(6) $$\beta =\frac{\eta }{\omega }$$

where η is the mechanical loss factor that is derived by η=2ζ, where ζ denotes the damping ratio that is frequency independent. For hysteretic damping model, damping ratio ζ is decided by the material mechanical quality factor $Q_{m}$ which can be derived by the following equation

(7) $$\zeta_{pzt}=\frac{1}{2Q_{m}}$$

For a harmonic motion, $x(t)=xe^{j\omega t}$, Equation (3) can be further expressed as

(8) $$M\ddot{x}+\left(1+j\eta \right)Kx=f\left(t\right)$$

With the variable stiffness multiplier and frequency independent damping ratio, the hysteretic damping model is capable of avoiding the overdamping problem for the modes that have natural frequencies larger than β/2. According to existing studies [32,45], initial hysteretic damping ratios have been selected for the finite element modelling. These ratios are further updated based on experimental results.

## 3. Sparse Regularization Based Electromechanical Impedance Technique for Damage Quantification

### 3.1. Measurement of Changes in Impedance Signatures

Model based EMI methods for damage identification have been investigated. The most commonly used measurements for model updating are the impedance/admittance magnitudes around the resonant peaks in an impedance/admittance curve. However, the difficulty to ensure the high accuracy of the predicted impedance/admittance amplitudes at all the selected frequencies would limit the performance of damage identification with those magnitudes of impedance measurements for model updating. Since the structural damage causes significant frequency shifts at the resonance peaks of impedance responses [46], using the frequency shifts as the impedance measurements is expected to be more prominent for damage identification. It should be noted that, consequently, the amount of available measurement data, e.g., frequency shifts used for damage identification, will be reduced, which makes identification an indeterminate inverse problem. A detailed solution for addressing this challenge is demonstrated in the following sections.

### 3.2. Inverse Problem in EMI-Based Damage Identification

Many studies on the sensitivity analysis based methods have been carried out for structural damage detection. For damage identification, simulated and measured results are compared and structural damage indicators are estimated by minimizing the difference between them. In general, structural damage can be simulated as stiffness reduction [47]. In this study, the damage of the host structure is identified as elemental Young’s modulus reduction. 

The high working frequency used in EMI based methods ensures the sensitivity of using such methods to detect minor changes in host structures. Although the performance of using finite element modelling for the prediction of the high-frequency impedance response has been demonstrated by other researchers [29,45], a finer meshing is necessary to achieve the accurate simulation due to the small wavelength corresponding to the excitation frequency. Therefore, a large number of unknown parameters of segments or elements of the host structure have to be identified. On the other hand, the changes in structural parameters normally manifest themselves around the resonance peaks in the impedance signature in very limited numbers of measurements. Under such a situation, the identification problem becomes a highly underdetermined inverse identification issue. The significance of this study is represented by using a sparse regularization technique to solve the underdetermined inverse problem and accurately identify the stiffness reduction in plate structures. 

Assuming that the local structural damage is defined as a change in the structural parameter, i.e., Young’s modulus, the relationship between impedance responses and the structural physical parameters of the host structure can be expressed as

(9) $$\left[S\right]\left\{\Delta p\right\}=\left\{\delta_{f}\right\}$$

where {Δp} is the structural damage vector which can be defined as a ratio of the changes in structural parameters. When $\Delta p_{j}=0$, there is no change in the *j*th element of the host structure indicating that the element is intact. When $\Delta p_{j}=1$, this means that the *j*th element is completely damaged. $\left\{\delta_{f}\right\}$ is the difference in the resonance frequencies of impedance responses between the numerical predictions and experimental measured results. [S] is the sensitivity matrix of the resonance frequencies of impedance responses with respect to structural parameters. In this study, minor structural damage is simulated as a small drilled hole which can be expressed as the local stiffness reduction in a specific element or region of the plate. 

The proposed EMI based damage quantification approach uses the resonance frequency shifts of the measured impedance responses as the targets for updating the numerical model by matching the analytical values calculated from the finite element model to the measured ones through the least squares method as

(10) $$\delta_{f}={\left[S^{T}S\right]}^{-1}S^{T}\Delta p$$

### 3.3. Conventional Tikhonov Regularization Method

Like most of the inverse problems, Equation (10) is an ill-conditioned problem due to the limited number of impedance measurements, but a large number of system parameters of host structure to be updated. To provide bounds to the solution and stabilize the inverse problem, the damped least-squares (DLS) method is widely used. The DLS solution of Equation (10) can be derived as

(11) $$\delta_{f}={\left[S^{T}S+\lambda I\right]}^{-1}S^{T}\Delta p$$

where λ is the non-negative damping coefficient governing the participation of least-squares error in the solution.

Equation (11) can be solved using the conventional Tikhonov regularization method by minimizing the following objective function

(12) $$J\left(\left\{\Delta p\right\},\lambda \right)=\left\|[S\right]\left\{\Delta p\right\}-{\left\{\delta_{f}\right\}\|}_{2}^{2}+\lambda \|{\left\{\Delta p\right\}\|}_{2}^{2}$$

where $\|\;{\|}_{2}$ denotes the *l*_2_ norm, and the second term provides restraint to the solution. The second term of the right part of Equation (12) is the regularization term for the stabilization of the damped least squares solution $\left\{\Delta \tilde{p}\right\}$. 

Theodoridis, et al. [48] pointed out that the solution of Tikhonov regularization method for the highly underdetermined ill-condition problems is over smoothed. Therefore, the solutions tend to distribute to several more elements around the damaged element rather than damaged element only. It could cause non-negligible errors during the structural damage identification process since the real structural damage usually concentrates in a few locations at the early stage. To obtain the more accurate sparse solutions in structural damage quantification of the true circumstance, the sparse regularization technique is applied in this study, instead of using the conventional Tikhonov regularization method.

### 3.4. Sensitivity Based Damage Detection Method Using Sparse Regularization

In general, only the impedance response in a certain frequency range can be used in the EMI based method owing to the computational demand. Therefore, a very limited number of frequency shifts can be extracted from the impedance measurements in the selected frequency range. According to Park, et al. [10], multiple frequency ranges containing 20 to 30 peaks are normally selected for damage evaluation due to the fact that a higher density of modes implies a larger dynamic interaction. In the practical model updating based EMI technique, simulating the impedance signature in a higher frequency range will also significantly increase the computational demand and decrease the computational efficiency, since it requires a much smaller element size for a finer mesh. Therefore, the inverse identification problem will be highly underdetermined. To solve such inverse problems and impose the sparsity regularization term as a restriction for the solution, Equation (12) can be rewritten as

(13) $$J\left(\left\{\Delta p\right\},\lambda \right)=\left\|[S\right]\left\{\Delta p\right\}-{\left\{\delta_{f}\right\}\|}_{2}^{2}+\lambda \|{\left\{\Delta p\right\}\|}_{P}^{P}$$

where the sparse regularization term $\|{\left\{\Delta p\right\}\|}_{P}^{P}$ with 0≤P≤1 is used to enforce the solution being sparse [49]. *l*_1_ norm regularization is applied in this study, since the *l*_1_ norm regularized optimization is a convex problem which can be solved by more efficient linear programming techniques [50,51]. Zhang, et al. [52] used *l*_1_ norm regularization for structural damage identification with vibration measurements. Then, the inverse problem is solved by minimizing the following equation based on *l_1_* norm regularization

(14) $$J\left(\left\{\Delta p\right\},\lambda \right)=\|\left[S\right]\left\{\Delta p\right\}-{\left\{\delta_{f}\right\}\|}_{2}+\lambda \|{\left\{\Delta p\right\}\|}_{1}$$

where $\|\;{\|}_{1}$ denotes the *l*_1_ norm. The solution of Equation (15) can be obtained by solving the least absolute shrinkage and selection operator (LASSO) problem, which can be expressed as

(15) $$\mathrm{Minimize}\;\|\left[S\right]\left\{\Delta p\right\}-{\left\{\delta_{f}\right\}\|}_{2}\;\mathrm{subject}\;\mathrm{to}\;\|{\left\{\Delta p\right\}\|}_{1}\leq t$$

**Figure 1:**
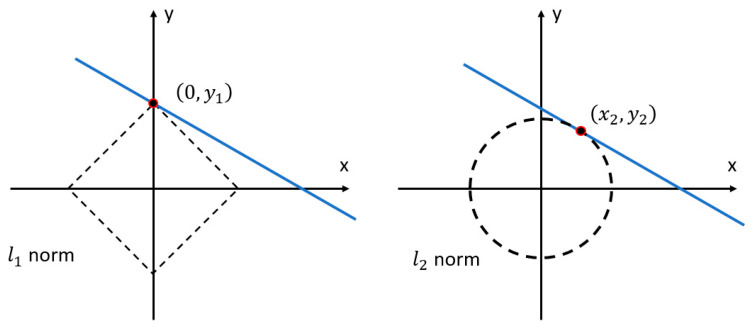
*l*_1_ and *l*_2_ norm solutions for the linear underdetermined problem.

The only difference between using Equations (12) and (14) to solve the ill-condition inverse identification problem is the regularization term. The *l*_1_ norm regularization term $\|{\left\{\Delta p\right\}\|}_{1}$ is used in the objective function, instead of the *l*_2_ norm regularization term ${\left\|\{\Delta p\right\}\|}_{2}$ in the conventional Tikhonov regularization. The intuitive explanation of the sparsity of the *l*_1_ norm regularization is shown in Figure 1. As shown in Figure 1, the dashed lines denote to the constraint regions of using *l*_1_ and *l*_2_ norm regularization, that are a square and a circle, respectively. The possible solutions are expressed as the blue straight line which is tangent to the restraint region. It is clearly observed that the solution using *l*_1_ norm regularization is most likely to be obtained in the corner of the constraint region, which is located on the axle with one of the coordinates equal to zero (e.g., $x=0,\;y=y_{1}$). In contrast, the Tikhonov regularization method is more likely to obtain the solution with both nonzero coordinates which is not sparse ($x=x_{2},\;y=y_{2}$). 

**Figure 2:**
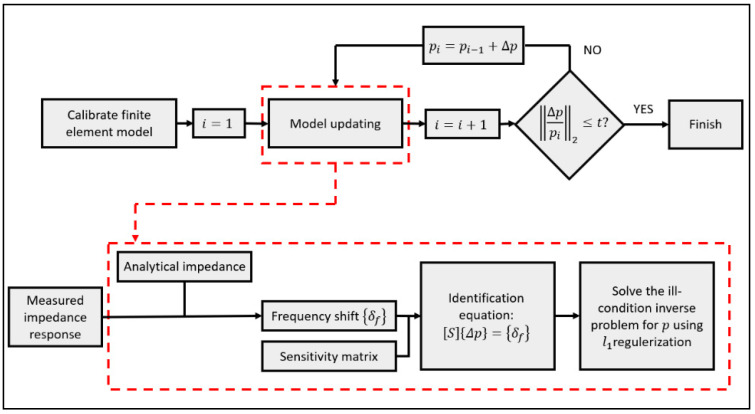
The flowchart of the proposed damage quantification approach

The flowchart of the proposed approach for structural damage quantification of plate structures by using sparse regularization based impedance technique is demonstrated in Figure 2. For the flowchart of the proposed damage quantification approach, the first task is to simulate the undamaged host structure with a surface bonded PZT sensor. The initial parameters used in this baseline model were calibrated and updated based on the measured impedance responses from the intact structure. The second task is to update a set of unknown structural parameters of the host structure which represent the elemental stiffness parameters by comparing the measured and analytical impedance responses, until the difference between them meets the convergence criterion. In this step, frequency shifts between the measured and analytical impedance resonance frequencies were used for model updating. The *l*_1_ regularization technique was used to solve the ill-conditioned inverse problem which is expressed in Equation (14). It is noted that the sensitivity matrix should be re-calculated in every iteration, when the stiffness parameters are updated. In this study, the convergence criterion is defined as

(16) $$\|{\frac{\Delta p}{p_{i}}\|}_{2}\leq t$$

where i denotes the $i_{th}$ iteration of the model updating process, Δp is the increment for the structural stiffness parameters. The limitation of convergence criterion t is defined as 0.0001.

## 4. Experimental Studies

Experimental studies on an aluminium plate structure installed with surface bonded PZT transducers have been conducted. Firstly, the baseline impedance responses from the intact host structure were obtained for the calibration of the initial finite element model. The measured impedance responses from the damaged structure were then used for the damage identification using the proposed sparse regularization based structural damage quantification technique. The details of the experimental setup and the damage identification results are presented and discussed.

### 4.1. Introduction and Experimental Setup

**Figure 3:**
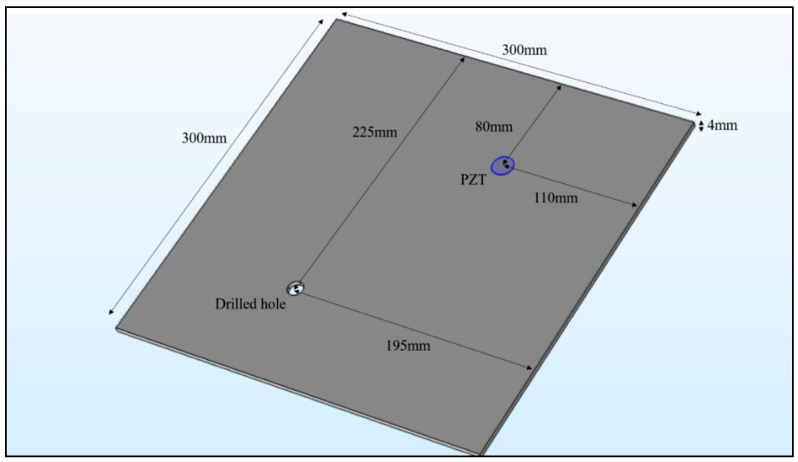
Damaged aluminium plate with a surface bonded piezoelectric (PZT) transducer.

To experimentally validate the accuracy of the built finite element model for the PZT-plate structure interaction and investigate the performance of the proposed damage identification approach, a 300 mm × 300 mm × 4 mm square aluminium plate was used as the testing specimen in this study, as shown in Figure 3. The dimensions of the selected PIC255PZT transducer was ∅8 mm×0.25 mm. The PZT transducer was bonded on the structural surface by commercial epoxy adhesive. The thickness of the bonding layer was kept as approximately 0.15 mm. The location of the installed PZT transducer and the introduced structural damage are shown in Figure 3. The PZT transducer was placed 80 mm and 110 mm away from the two edges of the aluminium plate, respectively. It should be noted, that the PZT transducer was placed at an asymmetric location on the specimen to ensure that any two structural damages with similar severity would not lead to the same impedance response. To ensure that the test results are repeatable and reliable, two similar specimens under the intact condition were adopted for the baseline test.

**Figure 4:**
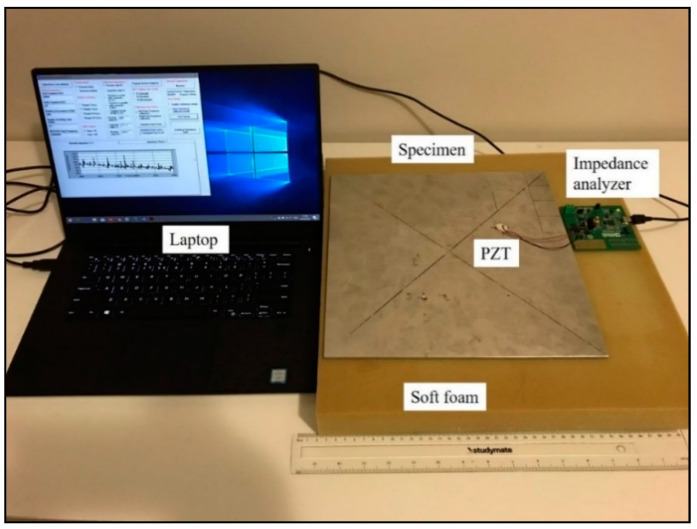
Configuration of the experimental test

In this study, damage was introduced as a hole created by a hand drill with a diameter of 6 mm. To locate the damage via the proposed approach, the aluminium plate was further divided into 100 square segments. Each square area has a size of 10 mm × 10 mm. In other words, there were 100 unknown parameters of the host structure in the inverse identification. The experimental configuration is shown in Figure 4. An impedance analyser EVAL-AD5933 was used for the actuation and sensing of the PZT transducer. The driving voltage of the PZT transducer was configured at 1 volt with a sweeping frequency range from 20 kHz to 30 kHz. To provide the free boundary condition, the specimen was placed on soft foam.

### 4.2. Model Calibration for Aluminium Plate with Surface Bonded PZT Transducer

To quantify the damage using the sensitivity based model updating with sparse regularization and resonance frequency shifts, the finite element model of the intact aluminium structure with a surface bonded PZT transducer was built. In this study, a three-dimensional finite element model was developed by using COMSOL Multiphysics 5.2, with eight-node solid elements. The cylinder PZT transducer was first modelled in COMSOL Multiphysics 5.2 using the property parameters in accordance with PIC 255, which are provided by the manufacturer, as given in Table 1. The properties of the aluminium plate and bonding layer from the manufacturer are listed in Table 2. The hysteretic damping model was adopted to define the damping of the PZT transducer, bonding layer and host structure. The parameters from manufacturers were initially used for the simulation and then updated based on the experimental testing results. In order to achieve the accurate prediction of impedance responses for damage quantification, numerical model of the plate structure with a surface bonded PZT transducer was calibrated by using the experimental results. The Piezoelectric Device toolbox in COMSOL Multiphysics was selected to simulate the highly complex relationship between solid mechanics and electrostatics for the piezoelectric materials.

**Figure 5:**
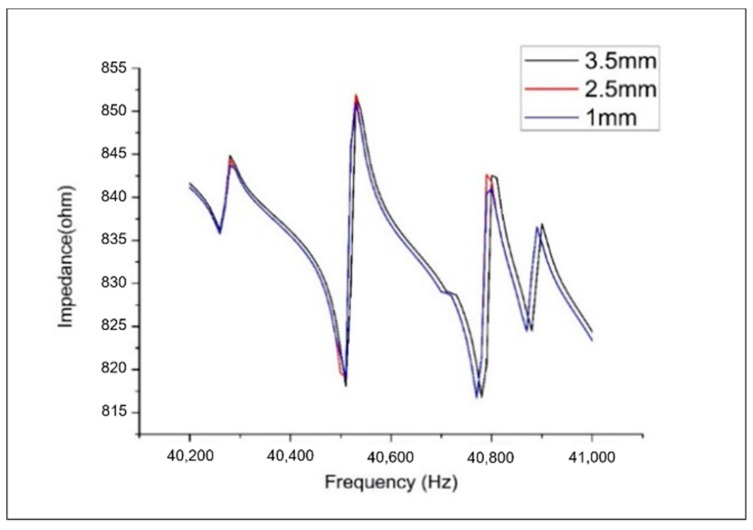
Convergence analysis of the numerical finite element model to calculate impedance responses with different mesh sizes.

Based on studies by Makkonen, et al. [53], three to five nodal points for each half wavelength are necessary to ensure a sufficient accuracy for the prediction. In this case, the wave velocity travelling in the aluminium plate can be calculated as $c=\sqrt{\frac{E_{Al}}{\rho_{Al}}}=5104.1\left(m/s\right)$, where E and ρ denote the elastic modulus and mass density of the aluminium material, respectively. For the selected impedance frequency range from 20 kHz to 30 kHz, the sufficient element size was estimated as 2.5 mm for the minor structural damage identification. The convergence analysis was conducted to obtain the mesh size for an accurate impedance response calculation. It can be observed from Figure 5 that convergence is reached at the eight-node hexahedron element size of 2.5 mm in the high frequency range from 40,200 Hz to 41,000 Hz. Thus, it could be concluded that the 2.5 mm mesh size of the eight-node hexahedron element is sufficiently fine for conducting the PZT-structure interaction modelling and is as efficient as possible for impedance computation. 

**Figure 6:**
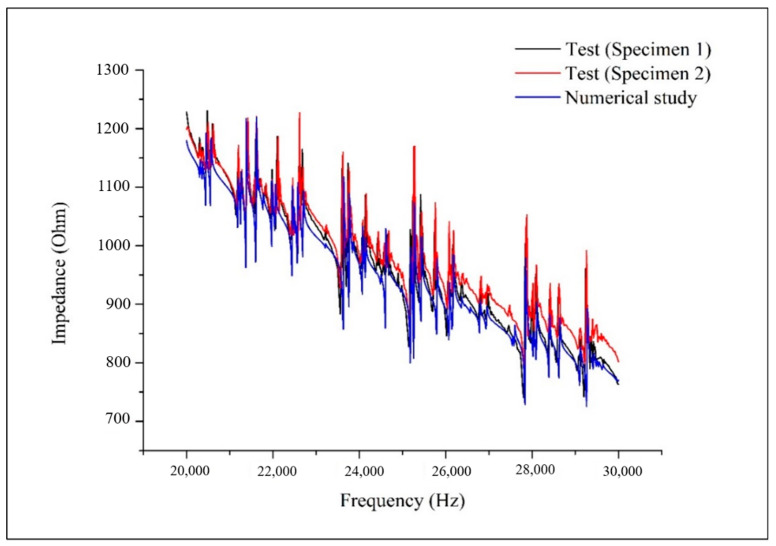
Comparison of the impedance responses from test specimens and the developed coupled finite element model.

Based on the sensitivity analysis of the resonance frequencies with respect to the system parameters, the most sensitive parameters were selected to be updated. The modified parameters are shown in Table 3. Figure 6 indicates that the developed coupled PZT-plate structural finite element model provides reasonable simulation results to well match with the experimental testing results of the impedance responses. It should be noted that the prediction of the resonance frequencies completely matches the test results. In other words, the outcome has a sufficient accuracy for damage identification using the impedance sensitivity based method with resonance frequency shifts in impedance responses. The remaining difference between the magnitudes of the numerical results and the testing results could be caused by manufacturing errors or other uncertainties. However, the imperfection would not influence the performance of the proposed approach, as only the frequency shifts of resonance peaks are defined as the measurements of impedance response changes, which will be used for the subsequent structural damage quantification.

### 4.3. Damage Quantification in a Plate Structure

For finite element modelling, many possible failure modes and damage phenomena can be simulated as changes in the bending stiffness of the elements [47]. In this study, minor structural damage in the aluminium plate is simulated as a reduction in the Young’s modulus of the segments. In this case, the formulation of the inverse identification problem in Equation (10) can be represented as

(17) [S]{ΔE}={Δf}

where {ΔE} denotes the vector of Young’s modulus changes in each segment of the host structure, {Δf} represents the resonance frequency shifts in the impedance responses from the undamaged and damaged plate structural models, and [S] is the sensitivity matrix of the resonance frequency shifts with respect to the Young’s modulus of the segment of the host structure. Then, the Young’s modulus of a specific segment of the host structure can be presented as

(18) $${\bar{E}}_{i}=E_{initial}\left(1+\eta_{i}\right)$$

where $\eta_{i}$ (0≤η≤−1) is the damage factor of the $i^{th}$ segment. In this case, η=0 denotes that the segment is under the intact condition, while η=−1 denotes that the segment is completely damaged. The sparse regularization technique has been used for structural damage identification with vibration measurements, and the complete procedure of this method is described by Van den Berg and Friedlander [54]. The identification results by using sparse regularization will be compared with those obtained from using the Tikhonov regularization method. 

In this study, the frequency shifts of 28 resonance peaks in the impedance responses between 20 kHz and 30 kHz were selected for damage identification. In the first step, the host structure was divided into 100 square segments of equal size, and the Young’s modulus changes in these segments were identified. The damaged segments were then further divided into nine square segments. The stiffness changes of these sub-segments were calculated in order to acquire the precise location and severity of the structural damage. 

**Figure 7:**
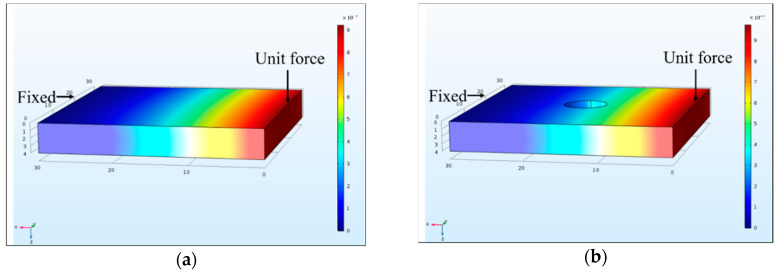
Finite element model for the equivalent Young’s modulus change (30 mm × 30 mm segment): (**a**) intact element; (**b**) damaged element.

**Figure 8:**
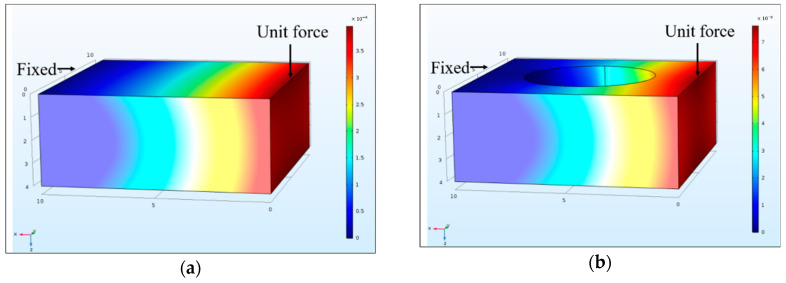
Finite element model for the equivalent Young’s modulus change (10 mm × 10 mm segment): (**a**) intact element; (**b**) damaged element.

It should be noted that structural damage is simulated in a different form compared with real experimental tests. Specifically, the Young’s modulus of each segment was used for model updating in the proposed damage identification method, but the actual structural damage was introduced as a drilled hole. It is necessary to estimate the equivalent Young’s modulus change corresponding to the drilled hole in the damaged segment. According to Li, et al. [55], the damage effect on the structure is equivalent to a bending stiffness reduction in the whole segment. Thus, the Young’s modulus changes of the drilled plate segment can be approximately estimated by the displacement method in the finite element analysis. The equivalent stiffness changes can be calculated based on displacements along the axis of the thickness direction produced by a unit force. The finite element models for the calculation of the equivalent Young’s modulus change are shown in Figure 7 and Figure 8. In this numerical study, the segments are fixed on one side, and a unit force is applied on the free side of the segment. The analytical stiffness reductions of the same damage in a 30 mm × 30 mm segment and a 10 mm × 10 mm segment are 5.32% and 48.9%, respectively. These theoretical values are considered as the approximate stiffness reductions caused by the drilled hole damage in experimental studies.

### 4.4. Identification Results and Discussion

In this section, experimental tests on the undamaged and damaged plates were conducted and the identification results were obtained to validate the accuracy and effectiveness of the proposed approach. The experimental studies were conducted to locate and quantify the severity of the structural damage in the aluminium plate. The configuration of the host structure and surface-bonded PZT transducer is introduced in Section 4.2. Frequency shifts of the resonance peaks in impedance curves were used as the impedance measurements. More than 30 resonance peaks were obtained in the impedance curve between 20 kHz to 30 kHz. Considering the errors between the analytical prediction and the measured results from the experimental test, 28 of these resonance peaks were selected for damage quantification. The peak frequencies obtained from the undamaged structure, damaged structure and finite element model under the intact state are compared in Table 4. The predictions of the finite element model are sufficiently accurate for damage identification. 

**Figure 9:**
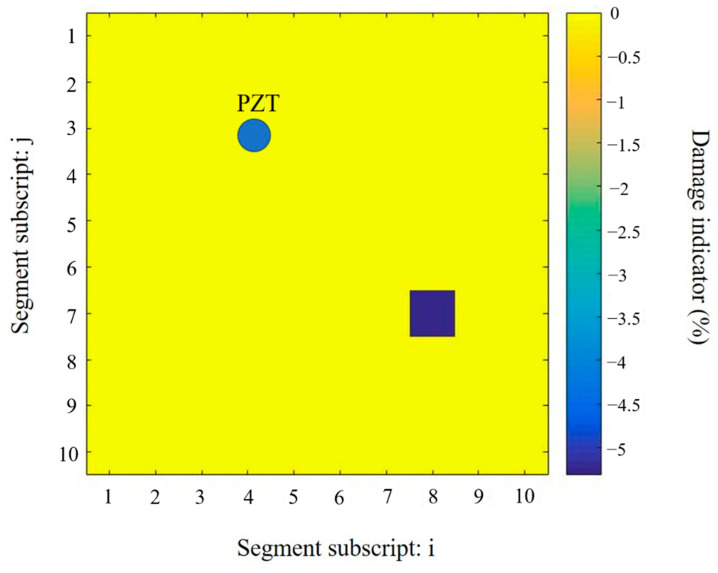
Location and severity of the actual damage.

A single damage scenario is considered in this section. There were 100 potential damage locations on the thin aluminium plate. Figure 9 shows the numbering of these segments. The subscripts i and j are used to define the segment, for example, segment $D_{4,3}$ is where the PZT transducer was bonded. The actual location and severity of the damage are presented in Figure 9. The actual damage is located in the segment $D_{7,8}$. The damage indicator is defined as $\eta =\frac{\left(E_{d}-E\right)}{E}$, where E and $E_{d}$ denote the Young’s modulus of the segment under the intact and damaged states, respectively. The severity of the drilled hole is equivalent to the Young’s modulus decrease, which is estimated by the displacement method in the finite element analysis as described above in Section 4.3. 

**Figure 10:**
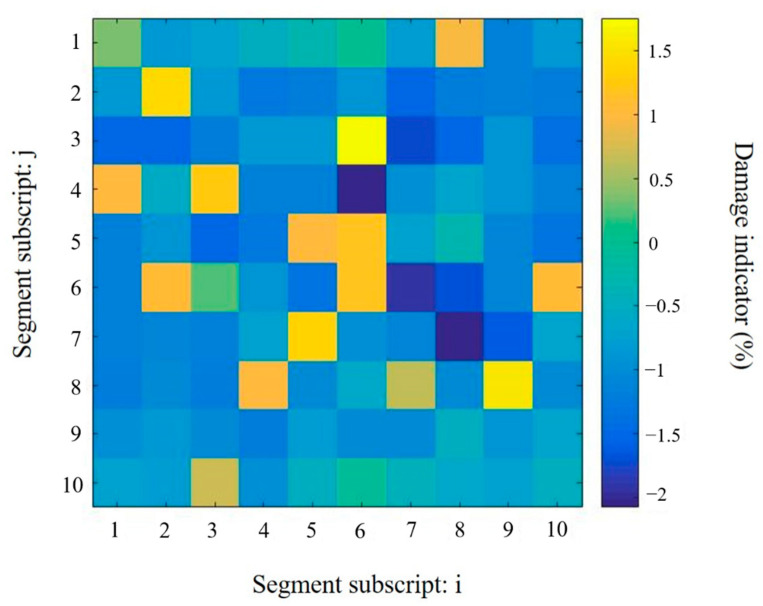
Damage identification results in the plate obtained using model updating with Tikhonov regularization method.

Figure 10 shows the damage quantification results by using the frequency shifts of the resonance peaks listed in Table 4 with the Tikhonov regularization method. In the solution using Tikhonov regularization, most of the elements tend to be non-zero, which means that the solution is not sparse. A number of false predictions are observed in the identification results. The convergence solution has been achieved by the proposed Tikhonov regularization method in two iterations. In the actual damaged segment $D_{7.8}$, the identified damage severity is −2.067%, which is less than half of the real Young’s modulus reduction in the damaged segment. The false predictions in $D_{4.6}$ and $D_{6.7}$ are approximately −1.7%. It is hardly possible to exclude them from the damaged segments without any prior knowledge. The identification results, as shown in Figure 10, fail to accurately indicate the correct location of the structural damage.

**Figure 11:**
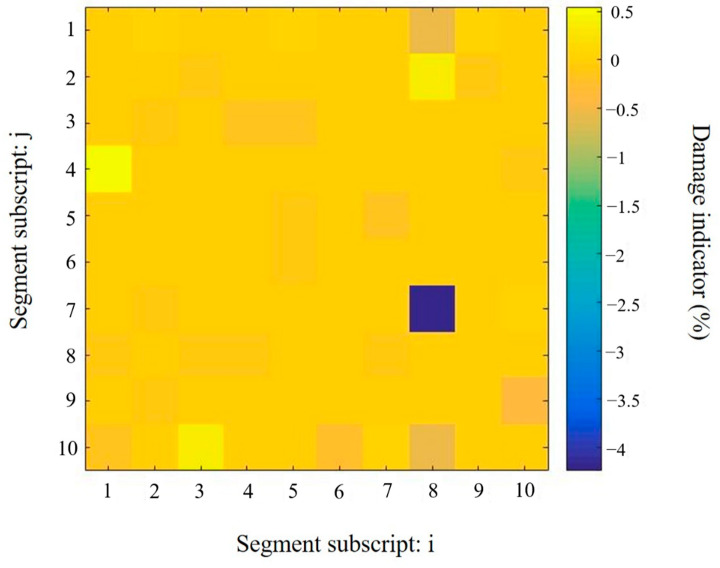
Damage identification results in the plate obtained using the proposed approach with sparse regularization.

In contrast, the identification results of using the proposed approach with sparse regularization, as shown in Figure 11, clearly indicate the correct damage location and severity. After three iterations, the convergence solution has been achieved by the proposed sparse regularization method. The predicted damage severity in the segment $D_{7.8}$ is −4.28%, which is close to the actual damage value of 5.32%. It should be noted that the Young’s modulus change corresponding to the actual damage is estimated. Thus, the identification result from the proposed approach can be regarded as a relatively accurate result. In addition, the false predictions in the undamaged segments are much lower than the prediction in the damaged segment. The maximum value is only −0.5% at only a couples of locations. The location of the introduced damage can be identified accurately.

**Figure 12:**
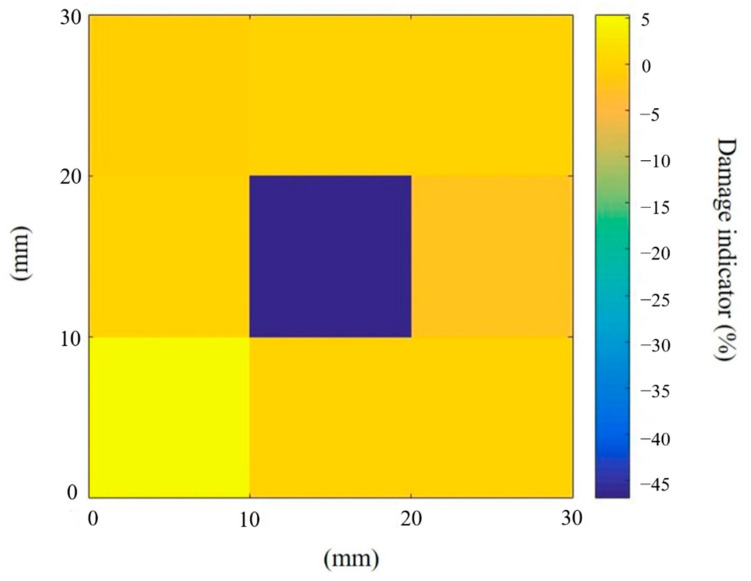
Damage identification results of the damaged segment.

To further identify the more accurate location and severity of the damage, segment $D_{7.8}$ was divided into nine square sub-segments, with each having a size of 10 mm × 10 mm. The identification results for the segment $D_{7.8}$ are shown in Figure 12. The location of the actual damage is identified at the centre of segment $D_{7.8}$. Using the proposed approach, the largest value of the identified damage is at the centre of this segment, with a stiffness reduction of −46.8%, as shown in Figure 12. There is only a relative error of −2.1% between the identified and obtained theoretical stiffness reductions.

## 5. Experimental Study on the Influence of Temperature Variation

Experimental studies were conducted to evaluate the performance of the proposed method under the effect of environmental uncertainty, i.e., the temperature conditions of the conducted tests and the results of these studies are presented in this section. The investigation focuses on the influence of temperature variation on the impedance signature. Experimental tests were carried out in a normal range of outdoor temperatures. Extreme temperature was not considered in this study.

### 5.1. Previous Studies on the Effect of Uncertainties on Impedance

Due to the nature of piezoelectric materials, the electrical property of a piezoelectric transducer is sensitive to variation in environmental conditions, such as temperature and humidity. For a system monitoring an actual outdoor structure, the temperature difference between day and night could lead to non-negligible changes in the impedance responses. Studies have been carried out to quantify the effect of temperature variation on the EMI based SHM technique. Since Park, et al. [56] investigated the significant changes in the electrical impedance of piezoelectric transducers due to varying temperature, efforts have been made to resolve the errors in the EMI-based SHM methods. Temperature variation causes both vertical and horizontal shifts in the electrical impedance signature [57]. Sun, et al. [58] proposed a function correlation analysis for compensation. However, based on experimental studies on bolted pipe, reinforced aluminium composites and precision parts, Park et al. [56] noted that the proposed correlation method would not work for the impedance signature with a small distortion. It has also been found that the real part of the impedance signature changes insignificantly with temperature. 

Most of the existing studies about the compensation methods to counteract the effect of temperature on the impedance signature are based on the non-model based EMI technique using statistical damage indicators, such as RMSD. For the model-based EMI technique, the impact of temperature variation on the performance of damage identification has rarely been studied. However, the changes caused by the environmental uncertainty could lead to significant errors and false predictions. 

### 5.2. Temperature Effects on the Impedance Responses of a Plate Structure

In this study, the changes in the impedance signature caused by varying temperature are investigated. Tests were carried out based on the experimental configuration introduced in the above sections. In the experimental study of damage detection for an aluminium plate, the undamaged and damaged specimens were placed in the laboratory at 23.5 °C, as a normal daytime temperature. The temperature was measured both with a thermometer and the temperature module on the EVAL-AD5933 impedance analyser board. This state is considered as the reference state of measurement. Then, the specimens were placed in an outdoor condition at night-time at a temperature of 15 °C. To test the tolerance of the proposed approach for damage quantification, the finite element model was calibrated using the experimental results obtained from the laboratory temperature condition at 23.5 °C. Then, the damaged specimen was placed in the outdoor environment. The identification results are based on the impedance signature acquired from the damaged specimens at a temperature of 15 °C. The identification results indicate the performance of the proposed approach under varying temperature conditions.

**Figure 13:**
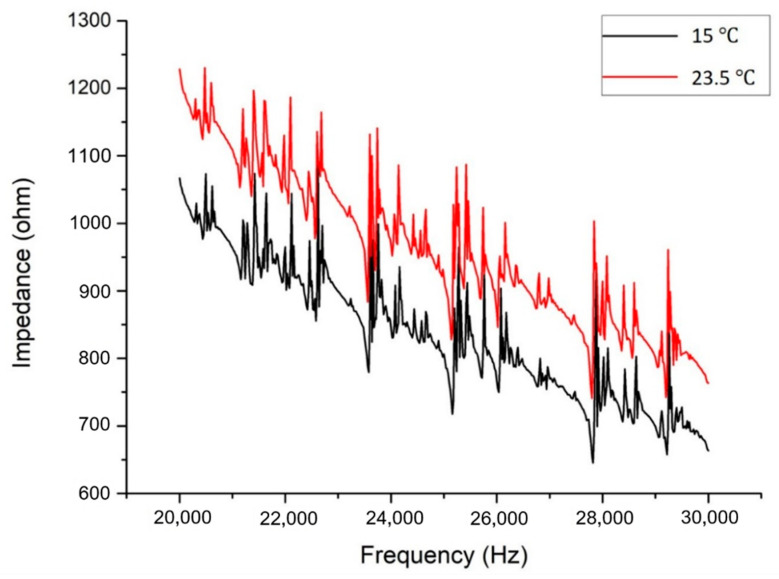
Impedance responses of an undamaged aluminium plate at different temperatures.

**Figure 14:**
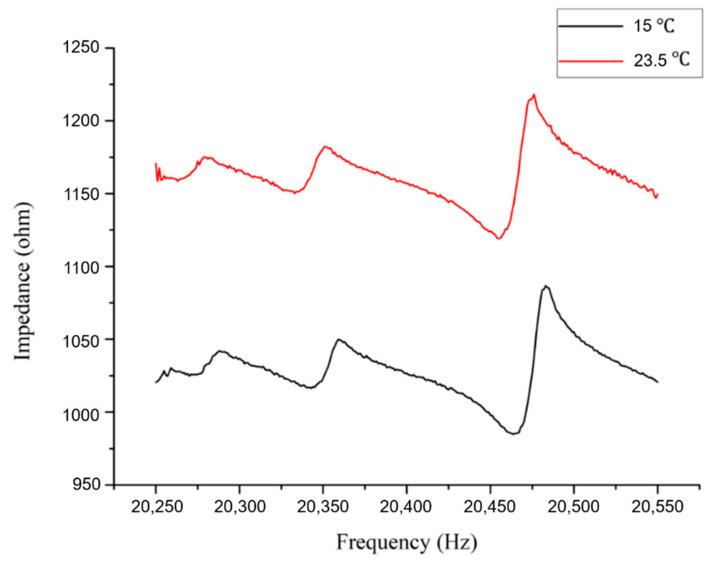
Impedance responses of a damaged aluminium plate at different temperatures.

Figure 13 illustrates the temperature effect on the impedance signatures of the PZT transducer bonded onto an undamaged plate specimen. In this case, decreasing temperature leads to a significant vertical shift in the impedance signature. It is worth noting that the horizontal shift is minor compared with that in the vertical direction. Meanwhile, the distortion in the impedance curves is negligible. For this reason, the selection of the resonance peaks for the proposed approach will not change significantly due to the temperature variation. Figure 14 shows the typical effect of temperature on the resonance peaks in the impedance signature of the damaged structure. Since the proposed approach only considers the horizontal frequency shifts of the resonance peaks, the effect of decreasing the temperature to 15 °C can be viewed as not bringing significant errors in the identification.

Table 5 lists the frequencies of the first three resonance peaks selected for the proposed approach for damage quantification in a plate structure. The change in resonance frequencies due to varying temperature is approximately 4 to 10 Hz, which is approximately 15% of the frequency shifts caused by the structural damage.

**Figure 15:**
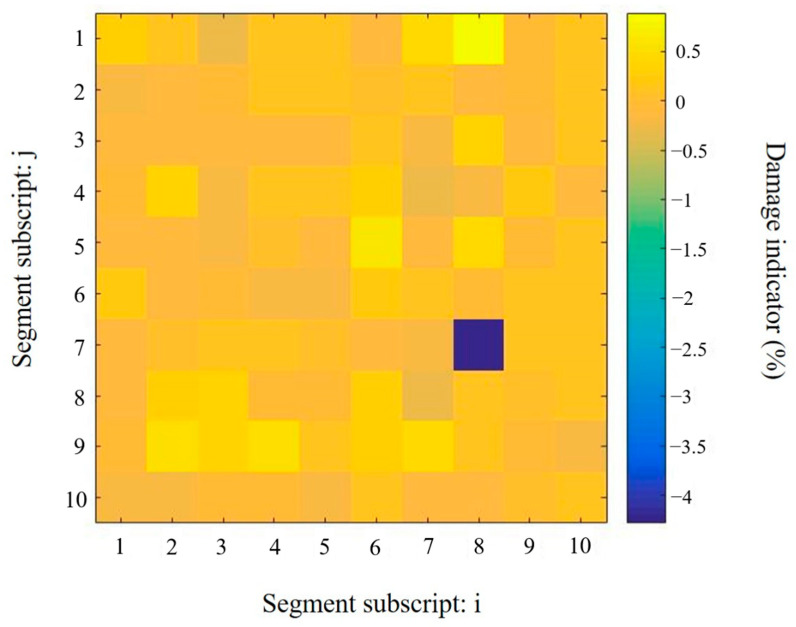
Damage identification results of the plate structure considering the temperature variation.

With an 8.5 °C difference in temperature, the damage identification results obtained using the proposed approach are shown in Figure 15. Without any prior information, the prediction correctly indicates the location of the drilled damage hole on the aluminium plate based on the impedance resonance frequency shifts. Even though the identified damage severity is lower than the prediction obtained using the impedance signature acquired from the reference temperature, with a decrease of 4.23% in the Young’s modulus, the value is still much larger than the predictions in the undamaged segments and close to the approximate actual damage severity, which is 5.32%. Compared with the results without temperature variation, a few more false predictions in the undamaged segments are observed. Once again, the values of these false predictions caused by environmental effects are much smaller than the identified true damage at the segment $D_{7,8}$.

## 6. Conclusions

This paper proposes structural damage quantification in plate structures with a model based EMI damage identification method. The study on the approach using resonance frequency shifts of impedance responses and the sparse regularization technique [44] has been extended to plate structures, which have been rarely researched by the model based EMI technique. An accurate finite element model was developed based on three dimensional finite element modelling of EMI simulations for plate structures. The accuracy of the developed finite element model was verified by the testing results of the aluminium plate under the intact condition. Considering the number of potential locations of damage, only a small number of frequency shifts in the impedance response from a single PZT transducer were used for damage identification. The Tikhonov regularization method leads to many false identifications when compared to the proposed approach based on sparse regularization. The proposed approach gives accurate identification of structural damage location and severity with only a low level of false identifications. The effect of environmental uncertainty is also investigated in this paper. Experimental testing under different temperature conditions was conducted and the measured impedance responses were used for identification. The results demonstrate that the proposed approach is not significantly affected by the temperature variations in the testing conditions.

More studies are needed to improve the performance of the proposed method. A future investigation is warranted to compare different optimization methods for the sparse regularization method, such as Broyden–Fletcher–Goldfarb–Shanno (BFGS) algorithm, to improve the computational efficiency. Nevertheless, more case studies with different damage types, such as laminated composite plate debonding, should be carried out to demonstrate the applicability of model based EMI technique.

## Figures and Tables

**Table 1:** Piezoelectric properties ofPIC255.

Parameters	Symbols	Values	Unit
Density	ρ	7800	$\mathrm{kg}/m^{3}$
Hysteretic damping ratio		0.00625	
Compliance	$s_{11}$	$1.59\times 10^{-11}$	$m^{2}/N$
	$s_{12}=s_{21}$	$-5.699\times 10^{-12}$	$m^{2}/N$
	$s_{13}=s_{31}$	$-7.376\times 10^{-12}$	$m^{2}/N$
	$s_{22}=s_{33}$	$2.097\times 10^{-11}$	$m^{2}/N$
	$s_{23}=s_{32}$	$-7.376\times 10^{-12}$	$m^{2}/N$
	$s_{44}=s_{55}$	$4.492\times 10^{-11}$	$m^{2}/N$
	$s_{66}$	$4.59\times 10^{-11}$	$m^{2}/N$
Piezoelectric strain coefficients	$d_{31}$	$-1.853\times 10^{-10}$	m/V
	$d_{32}$	$-1.74\times 10^{-10}$	m/V
	$d_{33}$	$3.94\times 10^{-10}$	m/V
	$d_{24}$	$5.35\times 10^{-10}$	m/V
	$d_{15}$	$5.35\times 10^{-10}$	m/V
Electric permittivity	$\varepsilon_{11}$	1649	
	$\varepsilon_{22}$	1649	
	$\varepsilon_{33}$	1750	

**Table 2:** Property parameters of the aluminum plate and bonding layer.

Property	Symbol	Value	Unit
Density (aluminium)	ρ	2710	$\mathrm{kg}/m^{-2}$
Young’s modulus (aluminium)	Y	72	GPa
Poisson ration (aluminium)	ν	1.4	
Damping ratio (aluminium)	ξ	0.35	
Density (adhesive)	ρ	1000	$\mathrm{kg}/m^{-3}$
Young’s modulus (adhesive)	Y	1.4	GPa
Poisson ratio (adhesive)	ν	0.4	
Damping ratio (adhesive)	ξ	0.016	

**Table 3:** Modified parameters of piezoelectric material in finite element modelling.

Parameters (PZT)	Unit	Value(Manufacturer)	Value(Updated)
Density	$\mathrm{kg}/m^{3}$	7800	7760
Mechanical loss factor		80	65
Compliance	$10^{-12}{\;m}^{2}N^{-1}$		
S11		15.9	13.8
S33		20.97	21.70
S66		45.9	43.19
Piezoelectric strain coefficient	$10^{-10}{\;\mathrm{mV}}^{-1}$		
d31		−1.853	−1.74
Damping ratio (bonding layer)		0.016	0.022
Young’s modulus (aluminium)	$N/m^{2}$	72e9	70.8e9

**Table 4:** Comparison of frequency shifts obtained from test results and simulations.

Experimental Result		Finite Element Model (Hz)
Undamaged (Hz)	Damaged (Hz)	
20,317.4	20,280.4	20,317.1
20,383.8	20,349.7	20,386.3
20,497.2	20,475.2	20,512.6
21,207.2	21,202.4	20,127.2
21,269.8	21,233.6	21,274.4
21,395.2	21,360.1	21,384.7
21,613.9	21,589.6	21,617.4
21,820.3	21,786.2	21,811.6
21,980.1	21,955.5	21,981.4
22,107.9	22,076.4	22,098.3
22,444.7	22,419.0	22,449.8
22,601.7	22,558.7	22,594.5
23,221.4	23,206.3	23,220.0
23,640.5	23,610.1	23,640.2
24,076.8	24,051.9	24,079.9
24,143.9	24,132.2	24,144.6
25,191.1	25,188.4	25,192.7
25,276.9	25,262.0	25,274.3
25,444.6	25,411.5	25,444.6
26,177.0	26,158.4	26,180.1
26,371.2	26,366.2	26,371.7
26,800.5	26,778.7	26,800.0
26,971.9	26,950.4	26,968.2
27,850.1	27,824.3	27,850.0
28,110.3	28,084.9	28,114.1
28,402.2	28,390.2	28,406.4
29,118.3	29,111.5	29,118.7
29,180.1	29,137.6	29,180.4

**Table 5:** Frequency of the first three resonance peaks with temperature variation.

Test Result (Damaged) (Hz)		Finite Element Model (Undamaged) (Hz)
23.5 °C	15 °C	
20,280.4	20,288.3	20,317.1
20,349.7	20,357.5	20,386.3
20,475.2	20,483.1	20,512.6
